# 基于高分辨UPLC-TOF-MS探讨A549/DDP与A549细胞内源性小分子代谢差异

**DOI:** 10.3779/j.issn.1009-3419.2018.08.01

**Published:** 2018-08-20

**Authors:** 伟鹏 洪, 永华 赵, 霖 曹, 迪 曹, 钟祥 赵, 晶 金

**Affiliations:** 1 510006 广州，中山大学药学院 School of Pharmaceutical Sciences, Sun Yat-sen University, Guangzhou 510006, China; 2 441500 南漳，南漳县人民医院检验科 Clinical Lab, People' s Hospital of Nanzhang County, Nanzhang 441500, China; 3 510006 广州，广州中医药大学中药学院 School of Chinese Materia Medica, Guangzhou University of Chinese Medicine, Guangzhou 510006, China

**Keywords:** 肺肿瘤, 耐药性, 顺铂, 代谢组学, Lung neoplasms, Chemoresistance, Cisplatin, Metabolomics

## Abstract

**背景与目的:**

顺铂获得性耐药是非小细胞肺癌（non-small cell lung cancer, NSCLC）化疗中至关重要并且有待进一步解决的问题。近年来通过细胞培养获得肿瘤耐药细胞，并将其作为代谢组学研究对象，寻找差异代谢物，获得潜在生物标志物，可以有效地为临床研究和治疗提供参考。本研究旨在通过代谢组学分析获取与顺铂耐药性相关的代谢物信息。

**方法:**

培养NSCLC细胞A549与其顺铂获得性耐药细胞A549/DDP后进行代谢物提取，通过超高效液相色谱-飞行时间质谱法对两种细胞的内源性小分子进行代谢组学分析，获取代谢差异物。

**结果:**

通过数据分析处理，获得40种差异代谢物，主要涉及磷脂、脂肪酸、氨基酸和能量代谢相关代谢物。

**结论:**

A549/DDP细胞的耐药性可能由于细胞膜结构的改变以及相关代谢途径的变化而导致。

## Abstract (en)

**Background and objective:**

Cisplatin acquired resistance is a vital problem in the chemotherapy of non-small cell lung cancer, which needs to be further addressed. In recent years, obtaining drug resistant cells from cell cultivation and serving for metabolomics research to find differential metabolites and get potential biomarkers, is a good reference for clinical research and cancer treatment. This study aimed to obtain metabolite information related to cisplatin resistance through metabolomics analysis.

**Methods:**

Metabolites were extracted from A549 cells and cisplatin resistant A549/DDP cells, and ultraperformance liquid chromatography coupled with time of flight mass spectrometry was used to perform metabolomic analysis of endogenous molecules of the two cells and obtain metabolic differences.

**Results:**

Through data analysis, 40 metabolites were identified as differential metabolites, mainly involving phospholipids, fatty acids, amino acids and metabolites related to energy metabolism.

**Conclusion:**

The drug resistance of A549/DDP cells may be caused by the changes of cell membrane structure and related metabolic pathways.

肺癌是世界上最常见的恶性肿瘤之一，而非小细胞肺癌（non-small cell lung cancer, NSCLC）占其中的80%-85%^[1]^。含铂方案是NSCLC晚期化疗的标准方案，然而其治疗效果经常达不到令人满意的程度，其中最主要的原因是顺铂的耐药性^[2]^。因此，深入了解NSCLC顺铂耐药的机制，并根据此获得有效的治疗方法，已经成为肿瘤研究的热点之一。

代谢组学是系统生物学新兴起的一个分支，它可以通过考察生物体的代谢产物及其动态变化来研究其代谢调控网络。目前已经广泛应用于肿瘤研究，对其发生发展过程的代谢物进行定性和定量分析可以识别未知的代谢产物^[3]^。目前肿瘤标志物的代谢组学研究主要通过对患者的血液、尿液或者癌组织进行分析，但由于年龄、性别、饮食、环境等因素所造成的个体差异较大，对肿瘤及肿瘤耐药标志物的识别产生了极大的影响。因此以肿瘤细胞作为代谢组学研究对象，通过细胞代谢物的差异表达寻找潜在的特异性标志物，可以有效地避免个体差异与复杂性问题，已经成为一种常见的研究方法^[4]^。

本研究以NSCLC与其顺铂获得性耐药细胞为基础，基于超高效液相色谱-飞行时间质谱法（ultraperformance liquid chromatography coupled with time of flight mass spectrometry, UPLC-TOF-MS），并结合代谢组学数据处理方法，对两种细胞的代谢提取物进行处理及分析，为考察NSCLC顺铂耐药细胞与敏感性细胞的内源性小分子代谢差异，寻找与其耐药性相关的代谢组学特征，探究潜在的标志物，为顺铂耐药性的确证和治疗提供参考。

## 1 材料与方法

### 1.1 仪器与试剂

Triple-TOF^TM^ 5600+型三重四级杆飞行时间质谱（美国AB SCIEX公司）。超高效液相系统（日本Shimadzu公司）：LC-30AD高效液相色谱仪、SIL-30AC自动进样器、SPD-M20A检测器、CTO-20AC柱温箱。25 cm^2^细胞培养皿（美国Corning公司）。胎牛血清（美国Gibco公司）、DMEM（Dulbecco's modified eagle medium）高糖培养液（美国Hyclone公司）；甲醇、乙腈（HPLC级，美国TEDIA公司）。

### 1.2 细胞来源

人NSCLC细胞A549和顺铂获得性耐药细胞A549/DDP购自中国科学院上海细胞库。

### 1.3 细胞培养

A549、A549/DDP细胞在37 ℃、5%CO_2_的条件下，贴壁生长于含10%胎牛血清的DMEM高糖培养基中。细胞均培养于25 cm^2^培养皿中，每种细胞平行培养6份，长满之后用于提取代谢组分并检测。

### 1.4 细胞提取方法

取对数生长期的细胞，用1 mL冰磷酸缓冲液（PBS）清洗2遍，加入1.5 mL冰甲醇后，静置3 min，刮下细胞，吸出细胞碎片溶于15 mL离心管中，加入3 mL二氯甲烷、1.5 mL甲醇和2.7 mL灭菌水，涡旋5 min，4 ℃下6, 000 rpm离心10 min，分别吸取上层极性层和下层非极性层于干燥离心管中，真空干燥挥干，置于-20 ℃冰箱中保存直至进样分析。

### 1.5 液相条件

#### 1.5.1 酸性条件

流动相A：0.1%甲酸-色谱乙腈；流动相B：0.1%甲酸-水；色谱柱：Waters Acquity UPLCBEH Amide（2.1 mm×100 mm, 1.7 μm）；柱温：45 ℃；流速：0.4 mL/min；进样体积：5 μL；洗脱梯度为：0 min，1%；0.1 min，1%；7 min，70%；7.5 min，1%；10 min，1%；10.01 min，停止。

#### 1.5.2 碱性条件

流动相A：95%乙腈-水（10 mmol/L乙酸铵，pH 9）；流动相B：5%乙腈-水（10 mmol/L乙酸铵，pH 9）；色谱住：Waters Acquity UPLCBEH Amide（2.1 mm×100 mm, 1.7 μm）；柱温：45 ℃；流速：0.4 mL/min；进样体积：5 μL；洗脱梯度为：0 min，1%；4 min，46.5%；10 min，70%；10.5 min，1%；14 min，1%；14.01 min，停止。

### 1.6 质谱条件质谱参数设置

离子源：电喷雾离子源（ESI），正负离子模式；源喷雾电压（ISFV）：-4, 500 V；辅助加热气温度（TEM）：550 ℃；雾化气(Gas1)：55 psi；辅助气（Gas2）：55 psi；气帘气（CUR）：35 psi；解簇电压（DP）：90 V；碰撞能（CE）：35。一级质谱母离子扫描范围：100 Da-1, 200 Da；IDA设置响应值超过100 cps的8个最高峰进行二级质谱扫描，子离子扫描范围：50 Da-1, 000 Da；数据采集所用软件为Analyst TF 1.7 software（AB SCIEX, Foster City, CA）。

### 1.7 统计学分析

#### 1.7.1 数据预处理

UPLC-QTOF-MS采集的原始数据利用MarkerView软件（AB SCIEX公司，美国）进行峰识别、匹配、峰对齐、滤噪等预处理。具体步骤如下：将原始质谱数据导入软件后，首先进行参数设置，峰识别（peak finding）参数选择：最小保留时间（minimum retention time）0.01 min，最大保留时间（maximum retention time）10 min或14 min，最小质荷比宽度（minimum spectral peak width）30 ppm，最小保留时间宽度（minimum RT peak width）5 scans，噪波阈值（noise threshold）100。峰对齐（alignment）参数选择：保留时间容差（retention time tolerance）0.1 min，质荷比容差（mass tolerance）10 ppm。滤噪（filering）参数选择：去除在少于20个样本中出现的峰，最大峰数目8, 000。经处理后即可得到峰表，峰表主要显示代谢物的保留时间、m/z等信息，里面有大量缺失值，产生的原因主要有：不存在该峰、峰存在但其检测值过小以致于不能被正确识别、未在80%以上的同组样本中出现，故需对其按“80%”原则剔除，再进行峰面积的归一化处理，即完成对数据的标准化预处理。

#### 1.7.2 模型构建

利用SIMCA 14.1（瑞典Umetrics AB公司）软件对标准化的峰表数据进行多变量分析，数据经过Parato变换后，选用正交偏最小二乘判别分析（Orthogonal Projections to Latent Structures Discriminant Analysis, OPLS-DA）进行有监督的数据分析，再进一步通过S-plot及代谢物的VIP值（Variable Importance on Projection，反应离子贡献度）、VIP值置信区间筛选差异代谢物。

#### 1.7.3 生物标志物的鉴定

生物标志物的鉴定方法主要是根据误差范围内分子量及相应的二级碎片信息与MassBank、HMDB、Lipidmap、Metlin、Chemspider等网站以及LipidView^TM^ 1.2（AB Sciex, Foster City, CA）所提供的信息的匹配度确定最终结构和其所涉及的相关代谢途径。根据OPLS-DA模型中变量的VIP值进行筛选，变量值> 1被认为该变量对组间区分有贡献，同时对VIP > 1.0的代谢物进行单变量统计分析，*P* < 0.05的代谢物纳入差异代谢物范围，进行结构鉴定。

## 2 结果

### 2.1 原始谱图和数据

本实验应用超高效液相色谱-高分辨质谱联用仪（UPLC-QTOF-MS）对A549、A549/DDP细胞的代谢产物进行了检测分析，得到其原始图谱，图 1为A549和A549/DDP细胞非极性和极性代谢产物总离子流图。通过对比，发现无论是极性代谢层还是非极性代谢层，两组细胞的谱峰在0.5 min-1.5 min、2 min-5 min的数量均基本相似，但是有一些峰的强度存在明显差异。

**1:**
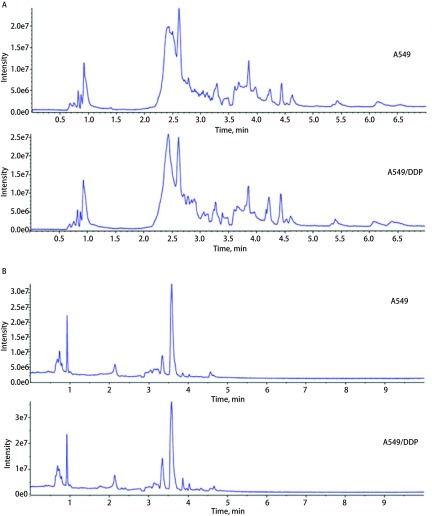
A549和A549/DDP细胞的非极性（A）和极性（B）层提取物的原始谱图（横轴表示时间，纵轴表示强度） The original spectrum of (A) non-polar metabolite profile and (B) polar metabolite profile of A549 and A549/DDP cells (X-axis stands for time, Y-axis stands for intensity)

### 2.2 A549和A549/DDP组细胞OPLS-DA分析

为考察A549和A549/DDP细胞之间的差异代谢情况，采用OPLS-DA对A549与A549/DDP细胞的极性代谢物层以及A549与A549/DDP细胞的和非极性代谢物层之间进行有监督的数据分析，获得OPLS-DA模型，如图 2所示。对于非极性代谢物模型（图 2A），包含3个主成分，其拟合参数分别为R^2^X=0.752、R^2^Y=0.998和Q^2^=0.967；而极性代谢物模型（图 2B）包含5个主成分，其拟合参数分别为R^2^X=0.928、R^2^Y=0.999和Q^2^=0.943，说明图 2A和图 2B模型的稳定性和预测率都比较高，适合于可靠解释A549和A549/DDP组细胞之间的代谢差异和发现两组之间的差异性表达代谢物。图 2A和图 2B模型中A549与A549/DDP细胞的极性代谢物层以及A549与A549/DDP细胞的和非极性代谢物层之间能完全分开，说明两组细胞之间有显著差异。图 2C和图 2D模型中表明两种细胞存在多种差异代谢物。

**2:**
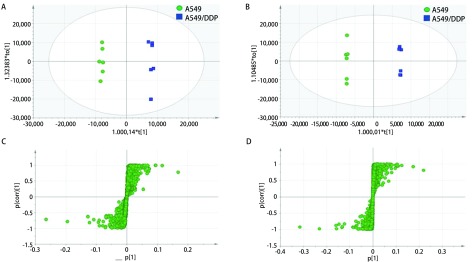
A549和A549/DDP细胞的非极性（A和C）和极性（B和D）代谢物的OPLS-DA模型图和OPLS-DA模型S-plot图 OPLS-DA results and S-plot results of non-polar metabolite (A, C) and polar metabolite (B, D) of A549 and A549/DDP cells

### 2.3 A549和A549/DDP组之间差异代谢物的鉴定

在A549和A549/DDP极性与非极性组细胞样品的S-plot图（图 2）中，代谢物点与原点附近的离子簇距离越远，则对样本类别分离的贡献越大，选择为潜在的差异代谢物，再进一步筛选出VIP值> 1和P值< 0.05的代谢物作为差异代谢物，并对这些差异代谢物进行结构鉴定，其中，非极性代谢物的鉴定结果见表 1，极性代谢物的鉴定结构见表 2。由表中结果可知，非极性差异代谢物主要为磷脂类成分，包含磷脂酰胆碱（phosphatidyl cholines, PC）、磷脂酰丝氨酸（phosphatidylserine, PS）、磷脂酰乙醇胺（phosphatidylethanolamine, PE）、磷脂酰肌醇（phosphatidylinositol, PI）和鞘磷脂（sphingomyelin, SM），五类成分中发现的差异代谢物各有11种、2种、5种、4种和6种；极性差异代谢物中主要包括胆碱、天冬氨酸类2种、组氨酸、柠檬酸、肉毒碱3种、泛酸、还原型和氧化型谷胱甘肽。

**1:** A549与A549/DDP细胞的非极性代谢差异物鉴定结果 Summary of the differential non-polar metabolites between A549 and A549/DDP cells

No.	RT (min)	m/z	Identification
1	8.35	677.49	PC (14:0/14:0)
2	10.06	691.54	PC (O-14:0/16:0)
3	8.44	703.51	PC (14:0/16:1)
4	9.38	705.52	PC (14:0/16:0)
5	11.16	719.58	PC O-32:0
6	9.48	731.54	PC (32:1)
7	11.55	761.59	PC 34:0
8	10.54	759.57	PC 34:1
9	11.25	771.61	PC O-36:2
10	2.58	495.33	LysoPC (16:0)
11	2.48	522.23	LysoPC (18:1)
12	11.38	701.53	PE (P-18:1/16:0)
13	9.81	715.51	PE (34:2)/PE (18:1/16:1)
14	10.82	717.53	PE (34:1)/PE (16:0/18:1)
15	10.87	743.55	PE 36:2/PE (18:1/18:1)
16	11.86	771.57	PE 38:2/PE (20:1/18:1)
17	10.82	767.54	PE (38:4)
18	10.37	789.54	PS (36:1)
19	9.45	791.56	PS (36:0)
20	9.46	702.56	SM 34:1;2
21	10.99	732.61	SM 36:0; 2
22	12.77	786.66	SM 40:1; 2
23	11.73	810.66	SM 42:3; 2
24	12.67	812.67	SM 42:2; 2
25	10.99	732.61	SM 36:0;2
26	9.28	862.56	PI 36:2/PI (18:1/18:1)
27	9.26	886.55	PI 38:4/PI (20:4/18:0)
28	9.68	888.57	PI 38:3/PI (20:3/18:0)
29	10.57	890.59	PI 38:2/PI (20:2/18:0)
PC, PC, LysoPC, PE, PS, SM and PI stands for phosphatidylcholine, lysophosphatidylcholine, phosphatidyl ethanolamine, phosphatidylserine, sphingomyelin and phosphatidylinositol respectively.			

**2:** A549细胞与A549/DDP细胞的极性代谢差异物鉴定结果 Summary of the differential polar metabolites between A549 cells and A549/DDP cells

No.	RT (min)	m/z	Identification	VIP
1	3.20	104.10	Choline	7.88
2	4.30	133.03	Aspartic acid	7.40
3	4.10	155.06	L-histidine	6.53
4	2.70	174.04	N-acetyl-L-aspartic acid	7.70
5	2.60	192.02	Citric acid	16.08
6	3.12	203.11	Acetylcarnitine	11.99
7	2.70	217.13	Propionylcarnitine	9.99
8	1.04	219.11	Pantothenic acid	7.84
9	2.50	245.16	2-Methylbutyroylcarnitine	3.72
10	4.40	307.08	Glutathione	13.91
11	5.40	612.15	Oxidized glutathione	12.38

## 3 讨论

通过UPLC-QTOF-MS的细胞代谢组学研究方法，对A549与顺铂耐药细胞A549/DDP进行培养及代谢相提取，并对两组细胞的内源性小分子代谢物进行分析，筛选并鉴定出了29种细胞非极性代谢差异物以及11种细胞极性代谢差异物。

在差异代谢成分中，发现多数磷脂类成分在A549和A549/DDP细胞中存在显著差异，主要包括PC、PS、PE、PI和SM。磷脂是构成细胞膜双分子层结构的基础，除了进行细胞内与外界的物质交换外，对细胞信号传导以及蛋白质功能也有重要的作用。而不同的细胞膜及其功能基本上可以从其分子结构上解释，这就与细胞膜中重要的磷脂组成部分的分子结构及所占比例密切相关。

通常情况下，耐药性细胞中的药物累积相对于敏感性细胞会比较低，其主要原因之一是药物流入的减少，而化疗药物一般都是亲脂性的。因此，药物流入的减少主要归因于细胞膜的生物和物理性质改变。耐药细胞通常有不同的脂质组成，这些变化改变了细胞膜的流动性、渗透性、结构、脂质填充密度、膜电位等基本性质，是导致穿过细胞膜流入细胞的药物减少的主要因素^[5]^。

除此之外，其他磷脂相关的生物效应同样对细胞耐药有重要的影响。胆碱和乙醇胺类磷脂作为其中含量最高最重要的两类磷脂，已有多项研究^[6-8]^表明其在肿瘤细胞中的含量以及其相关代谢酶的表达和活性与正常细胞有显著差异。磷脂酰丝氨酸是一种有免疫抑制作用的磷脂，通常存在于细胞的膜内层。它可以通过结合免疫细胞的磷脂酰丝氨酸受体，抑制其抗癌免疫效应和攻击效应，以此调控肿瘤细胞的生物进程以及恶性转化。在多种肿瘤细胞（包括乳腺癌、胶质瘤等）膜上都可以发现过量的磷脂酰丝氨酸^[9]^。而鞘磷脂则是脂质筏的重要组成成分，它可以直接参与跨膜蛋白的功能，并为多药转运蛋白提供最佳的脂质微环境。有研究者提出，肿瘤细胞膜上鞘磷脂含量的提高除了有助于脂质筏的延展之外，还可以招募多药耐药蛋白结合于脂质筏上，改变细胞膜的流动性，阻碍药物的摄取，致使细胞产生耐药性。而磷脂酰肌醇除了参与细胞膜的构成外，它在细胞中对于代谢调控、信号传导和细胞的各种生理功能起着非常重要的作用^[10, 11]^。因此，在该研究中发现与膜构成相关的几种重要磷脂成分（PC、PE、PS、SM和PI）均为两株细胞之间的差异代谢物，提示针对相应的磷脂代谢途径，可以改变细胞膜的性质，并影响所产生的信号传导和引起的生物效应，从而可能改善其耐药性。

除了磷脂类胆碱外，我们还发现在耐药细胞A549/DDP中其他胆碱成分与A549细胞相比同样也存在明显差异。早在20世纪末，科学家通过磁共振波谱分析就已经发现异常的胆碱代谢存在于多种肿瘤中，主要表现为高水平的磷脂酰胆碱和总胆碱。异常的胆碱代谢不仅参与肿瘤的增殖生长，还会促进其恶性转化。因此，胆碱类成分已逐渐被当做是肿瘤诊断和治疗的一种重要标志物，并且胆碱代谢的分子机制研究也成为抗肿瘤治疗研究的重点^[12, 13]^。已有研究^[14, 15]^在乳腺癌耐药（吉非替尼）细胞、前列腺癌耐药（卡铂）细胞和胰腺癌耐药（埃罗替尼）细胞中发现了其胆碱类代谢物的变化。因此，我们的实验结果说明胆碱代谢与NSCLC顺铂耐药性有潜在的相关性，通过调控其代谢通路可能会达到逆转其耐药性的作用。

除此之外，我们同样在两种细胞中发现了多种合成或代谢途径相关的差异代谢物。包括参与氨基酸合成代谢的天冬氨酸、组氨酸和泛酸，参与三羧酸循环和能量代谢的柠檬酸，参与脂质代谢和能量代谢的肉毒碱，以及参与磷酸戊糖途径、谷胱甘肽代谢的还原型和氧化型谷胱甘肽（glutathione, GSH）。通过查阅文献，我们发现本实验结果与既往研究存在一定的一致性。Schneider等^[16]^发现卵巢癌顺铂耐药细胞A2780-cis中的ATP基础水平与敏感性细胞A2780相比明显提高，并认为这是由于耐药细胞的能量代谢水平更旺盛的缘故导致，本研究中发现柠檬酸在两种细胞中存在明显差异，暗示了耐药细胞于敏感细胞的基础能量代谢可能有所不同。Lee等^[17]^通过代谢组学研究发现，膀胱癌细胞T24S与其顺铂耐药性细胞中的多种脂质有显著性差异，并提出脂质代谢重编程与顺铂耐药性密切相关的观点，因此肉毒碱作为脂质合成的基本分子，与顺铂耐药性的关系不言而喻。顺铂可以损伤细胞内线粒体，并使活性氧簇（reactive oxygen species, ROS）水平升高，导致胞内氧化应激，GSH是ROS的有效清除剂之一，而且GSH可以与顺铂形成复合物，因此细胞内GSH水平的变化是影响顺铂药效的潜在因素。现已有研究^[18, 19]^发现，抑制6-磷酸葡萄糖脱氢酶（glucose 6-phosphatedehydrogenase, G6PD）可以逆转卵巢癌，肺癌的顺铂耐药性细胞，G6PD催化反应所产生的的NADPH是生成还原性GSH的重要还原剂。此外，作为GSH代谢途径的主要上游调控基因，转录因子NF-E2相关因子2（Nf-E2 related factor-2, Nrf2）被发现对顺铂的耐药性也有影响^[20]^，说明谷胱甘肽等维持氧化还原稳态的分子是顺铂药效发挥的重要影响因素。而对于氨基酸的主要代谢物与顺铂的疗效的相关研究则较为缺乏，目前仅有研究^[21]^发现γ-氨基丁酸对于顺铂的肾毒性能发挥有效的缓解作用。因此这一方面的研究需要深入探讨。重编程一直是肿瘤靶向研究的热点，通过靶向不同肿瘤与正常组织或细胞相异的代谢通路，来达到抗肿瘤的目的并获取有效的治疗方法^[22]^。这种方法同样适用于解决肿瘤耐药的问题，通过影响代谢通路，使细胞的生理状态产生影响，使其对化疗药物更为敏感，不仅如此，化疗药的作用机制也可能部分通过代谢机制来完成，这可能更有利于增敏及逆转耐药。

综上，本实验通过对A549细胞及其顺铂耐药细胞A549/DDP的代谢组学分析，发现多数磷脂类成分以及部分代谢相关物质为两种细胞的差异代谢物，但其确证还需要进行进一步相关的体内外实验，希望能够为逆转NSCLC顺铂耐药性提供有效的参考和依据。
